# Supplementary material for: The Distinctive Serum Metabolomes of Gastric, Esophageal and Colorectal Cancers
Source: Cancers (Basel). 2021 Feb 10;13(4):720. doi: 10.3390/cancers13040720 (PMC7916516; doi:10.3390/cancers13040720)
Supplement: Supplementary file 1 [file cancers-13-00720-s001.pdf]

The distinctive serum metabolomes of gastric, esophageal and colorectal cancers

Supplementary File

Table S1. Differential metabolites (EC *vs*.non-EC) for esophageal cancer

Metabolite [48]	Metabolite [49]	Metabolite [50]	Metabolite *
Glucose↓			
Pyruvic acid↑			
Lactate↑			
Citrate↑			
A-ketoglutaric acid ↑			
Succinate↓			
Fumarate↑			
Malate↑			
Valine↓			
Histidine↑		Histidine↓	
Phenylalanine↓			
Threonine↓			
Leucine↓			
Methionine↓		Methionine↓	
Tryptophan↓	Tryptophan↓	Tryptophan↓	Tryptophan↓
Glutamine↑			
Glutamate↓			
Serine↓			
Glycine↓			
Alanine↓		Alanine↓	
Arginine↓			
Aspartate↑			
Cystine↑			
Proline↓		Proline↓	
Isoleucine↓			
Lysine↑			
Tyrosine↑	Tyrosine↓	Tyrosine↓	Tyrosine↓
Linoleic acid↓	Linoleic acid		Linoleic acid↑
Oleic acid↑		Oleic acid↑	Oleic acid↑
	Palmitoleic acid↑	Palmitoleic acid↑	Palmitoleic acid↑
	Choline↓		
Hypoxanthine↑	Hypoxanthine↓		
	Proline betaine↓		
	Indoleacrylic acid ↓		
	Isovaleryl-carnitine↓	Isovaleryl-carnitine↓	
	Inosine ↓		

	Alpha-Linolenic acid ↑		
	Piperine ↓	Piperine ↓	
	L-Octanoyl-carnitine ↓		
	Nonanoyl-carnitine ↓		
	Decadienoyl-carnitine ↓		
	Decanoylcarnitine↓		
	Docosahexaenoic acid ↓		
	Undecanoyl-carnitine ↓		
	Dodecenoyl-carnitine ↓		
	Phenylalanyl-Tryptophan ↓		
	Tetracosahexae-noic acid↓		
	Cortisol ↓		Cortisol↑
	Tetradecadien-carnitine ↓		
		3-hydroxy-butyrate↑	
Asparagine↓		Asparagine↓	
		Glycochenodeoxycholate↓	
		Glycocholate↓	
		Glycodeoxycholate↓	
		Hippurate↓	
		Mannose↑	
		Propionylcarnitine↓	
Pyroglutamine↓		Pyroglutamine↑	
		Threitol↓	
		10-heptadecenoate (17:1n7)↑	
Ribose↓			
Taurine↑			
Cis-Aconitic acid↑			
Hydroxyproline↓			
Ornithine↓			
Kynurenic acid↓			
Creatinine↓			
Urea↓			
Urate↓			
Monostearin↓			
Pipecolic acid↓			

Glyceric acid↓		
	10-nonadecenoate(19:1n9)↑<br>1-linoleoylglycerophosphocholine↓<br>1-linoleoylglycerophosphoethanolamine↓<br>1-myristoylglycerophosphocholine↓<br>1-oleoylglycerophosphoethanolamine↓<br>1-palmitoylglycerophosphoethanolamine↓<br>1-stearoylglycerophosphoethanolamine↓<br>2-hydroxydecanoic acid↓<br>2-methylbutyrylcarnitine (C5)↓<br>3-hydroxypropanoate↓<br>3-methyl-2-oxobutyrate↑<br>5-dodecenoate (12:1n7)↑<br>adrenate (22:4n6)↑<br>beta-alanine↓<br>catechol sulfate↓<br>creatine↓<br>deoxycarnitine↓<br>dihomo-linoleate (20:2n6)↑<br>docosadienoate (22:2n6)↑<br>docosapentaenoate (n3 DPA; 22:5n3)↑<br>docosapentaenoate (n6 DPA; 22:5n6)↑<br>eicosenoate (20:1n9 or 11)↑<br>ergothioneine↓<br>gamma-glutamylmethionine↓<br>gamma-glutamylphenylalanine↓<br>gamma-glutamyltyrosine↓<br>hexadecanedioate↑<br>indolelactate↓<br>indolepropionate↓<br>margarate (17:0)↑	

	myristate (14:0)↑<br>myristoleate (14:1n5)↑<br>N6-acetyllysine↓<br>O-methylcatechol sulfate↓<br>palmitate (16:0)↑<br>paraxanthine↓<br>pentadecanoate (15:0)↑<br>propionylcarnitine↓<br>ribulose↓<br>scyllo-inositol↓<br>stachydrine↓<br>stearate (18:0)↑<br>xylonate↓	
		Dodecanoic acid↓<br>Palmitic acid↑<br>LPA(18:1/0:0)↓<br>LysoPC(14:0/0:0)↓<br>LysoPC(18:2)↑<br>LysoPC(24:0)↑<br>LysoPC(18:4)↑<br>PC(14:1/P-18:1)↑<br>PC(16:0/18:2)↓<br>PC(24:1/22:6)↑

The arrows indicate reported increasing or decreasing trends relative to non-GC controls.

Table S2. Differential metabolites (CRC *vs.* non-CRC) for colorectal cancer.

Metabolite [44] N=24	Metabolite [51]	Metabolite [52]	Metabolite [55]	Metabolite [53]	Metabolite [54]
Glutamic acid			Glutamic acid↓	Glutamic acid	Glutamic acid↓
Phenylalanine		Phenylalanine		Phenylalanine	Phenylalanine↓
Alanine	Alanine	Alanine		Alanine	Alanine
Isoleucine	Isoleucine	Isoleucine			Isoleucine↑
Histidine				Histidine	
Serine					Serine↓
Lactic acid	Lactic acid	Lactic acid	Lactic acid↓		Lactic acid↑
Aspartic acid			Aspartic acid↓		
Succinate				Succinate	
Cysteine		Cysteine		Cysteine	Cysteine↓
Glutamine				Glutamine	
Leucine				Leucine	Leucine↓
Valine				Valine	Valine↓
Choline			Choline↓	Choline	
Fumaric acid			Fumaric acid		Fumaric acid↑
Tyrosine		Tyrosine		Tyrosine	Tyrosine↓
Asparagine				Asparagine	
Pyruvic acid			Pyruvic acid↑		Pyruvic acid↑
Arginine				Arginine	
Methionine				Methionine	
Tryptophan	Tryptophan			Tryptophan	Tryptophan↓
Acetoacetate					
Uracil					
Urea	Urea				Urea↓
Creatinine					Creatinine↑
Homovanillate				Homovanillate	
Guanidoacetate					
Putrescine					
	2,3-Butanediol				
	2-Amino-butanoic acid			2-Amino-butanoic acid	
	2-Hydroxy-butyricacid			2-Hydroxy-butyric acid	2-Hydroxy-butyric acid↑
	3-Hydroxy-butyric acid			3-Hydroxy-butyric acid	3-hydroxy-butyric acid↑
	3-Hydroxy-pyridine				
	Butanoic acid	Butanoic acid			
	D-Fructose	Fructose			Fructose↑
	D-Glucose		Glucose↑		Glucose↓
	D-Mannose		Mannose↑		

	Glycine	Glycine			Glycine↓
	Proline				Proline↑
	Uric acid			Uric acid	Uric acid↓
		Ethylene glycol			
		Stearic acid			
		Citric acid			Citric acid↑
		Lysine		Lysine	Lysine↓
		Inositol			Inositol↑
		Glyceric acid	Glyceric acid↓		
		Palmitic acid			
		Linoleic acid	Linoleic acid↑		
		2-Monostearoyl-glycerol	2-Monostearoyl-glycerol↓		
		Threonine			Threonine↓
		Glycerol-3-phosphate			
		Glyceryl palmitate	Glyceryl palmitate		
		Hexadecanoic acid			
		D-Ribose 5-phosphate		D-Ribose 5-phosphate	
		Glycerol		Glycerol	
		Nonane			
		Malonic acid	Malonic acid↓		
		Arabinose			Arabinose↑
		Threonic acid		Threonic acid	
			Hypoxanthine↓	Hypoxanthine	
			Xanthine↓		
			Tryptophyl-glutamate↓		
			3-methyl-2-oxobutyrate↑		
			5-oxoproline↓		
			Pseudouridine↑		
			Hydroxyiso-valeroyl carnitine↓	Hydroxyiso-valeroyl carnitine↓	
			Kynurenine↑		Kynurenine
			cis-4-decenoyl carnitine↑		
			Decanoyl-carnitine↑		
			γ-glutamylalanine↑		

	Heptanoate (7:0)↓	Heptanoate (7:0)↓	
	Aspartylleucine↓		
	1-stearoylglycerophosphoethanolamine↓		
	Theobromine↓		
	1-palmitoylplasmenylethanolamine↓		
	2-hydroxypalmitate↑		
	Phenylalanyl-glutamate↓		
	Leucylalanine↑		
	1-oleoylglycerophosphocholine↓		
	cysteine-glutathione disulfide↑		
	Paraxanthine↓		
	N-acetylglycine↑		
	Phenylalanylserine↑		
	glycolate ↓		Glycolate↑
	Octanoylcarnitine↑		
	Sucrose↑		Sucrose↑
	2-hydroxystearate↑		
	Theophylline↓		
	Ornithine↓	Ornithine	Ornithine↑
	3-dehydrocarnitine↓		
	Benzoate↓	Benzoate	
	Linolenate ↑		
	Uridine↓		
	Isovalerate↓	Isovalerate	Isovalerate↓
		Octanoic acid	Octanoic acid↑
		Decanoic acid	
		Gluconolactone	
		Gluconic acid	
		5-Amino-4-oxovaleric acid	
		Inosine	
		1-Methylnicotinamide	
		Myristoleic acid	
		Stachydrine	
		Isobutyryl-carnitine	

		Hydroxyproline	hydroxyproline↑
		3-Methyl-histidine	
		Perillic acid	
		2-oxovaleric acid	
		Ethylglutamine	
		Trimethylamine N-oxide	
		S-Methyl-cysteine	
		Cholic acid	
		Ethanolamine phosphate	
		Cysteine glutathione disulfide	
		Quinic acid	
		Indole-3-acetic acid	
		Sarcosine	Sarcosine↓
		B-alanine	
		Isobutyric acid	
		Betaine	
		Lauric acid	
		Ethanolamine	
		Citrulline	
			2-aminoiso-butyric acid↓
			2-amino-butyric acid↓
			3-hydroxy-isovaleric acid↓
			Phosphoric acid↑
			Nonanoic acid↓
			Threitol↑
			meso-erythritol↑
			2-ketoglutaric acid↑
			Xylose↑
			Arabitol↑
			Isocitric acid↑
			2-aminopimelic acid↑

	1,5-anhydro-glucitol↓<br>Sorbose↑<br>5-dehydroquinic acid↓<br>Hippuric acid↓<br>Galactose↓<br>Glucosamine↓<br>Glucuronic acid↑<br>Ascorbic acid↓<br>Palmitoleic acid↓<br>Elaidic acid↓<br>Maltose↑<br>Malic acid↑

The arrows indicate reported increasing or decreasing trends relative to non-GC controls.
